# Illuminating Polysulfide Distribution in Lithium Sulfur Batteries; Tracking Polysulfide Shuttle Using *Operando* Optical Fluorescence Microscopy

**DOI:** 10.1021/acsami.3c14612

**Published:** 2024-04-10

**Authors:** Kofi Coke, Michael J. Johnson, James B. Robinson, Alexander J. E. Rettie, Thomas S. Miller, Paul R. Shearing

**Affiliations:** † Electrochemical Innovation Lab, Department of Chemical Engineering, University College London, Torrington Place, London WC1E 7JE, U.K.; ‡ The Faraday Institution, Quad One, Becquerel Avenue, Harwell Campus, Didcot OX11 ORA, U.K.; § Advanced Propulsion Lab, UCL East, University College London, London E15 2JE, U.K.; ∥ Department of Engineering Science, University of Oxford, Parks Road, Oxford OX1 3PJ, U.K.

**Keywords:** Li−S batteries, lithium polysulfides, dendrites, in situ battery characterization, battery degradation, OFM, quantitative, electrolyte

## Abstract

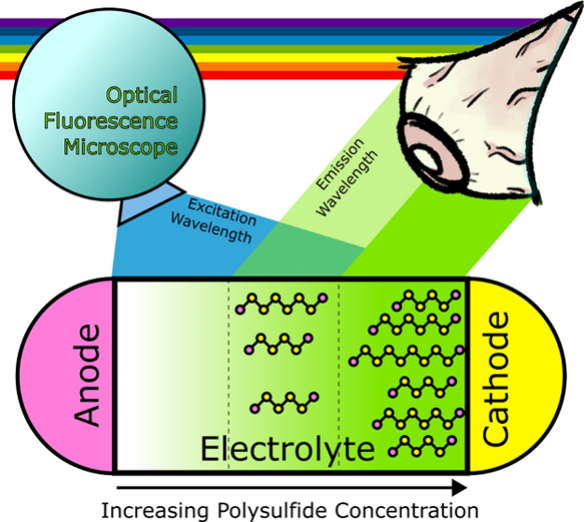

High-energy-density lithium sulfur (Li–S) batteries suffer heavily from the polysulfide shuttle effect, a result of the dissolution and transport of intermediate polysulfides from the cathode, into the electrolyte, and onto the anode, leading to rapid cell degradation. If this primary mechanism of cell failure is to be overcome, the distribution, dynamics, and degree of polysulfide transport must first be understood in depth. In this work, *operando* optical fluorescence microscope imaging of optically accessible Li–S cells is shown to enable real-time qualitative visualization of the spatial distribution of lithium polysulfides, both within the electrolyte and porous cathode. Quantitative determinations of spatial concentration are also possible at a low enough concentration. The distribution throughout cycling is monitored, including direct observation of polysulfide shuttling to the anode and consequent dendrite formation. This was enabled through the optimization of a selective fluorescent dye, verified to fluoresce proportionally with concentration of polysulfides within Li–S cells. This ability to directly and conveniently track the spatial distribution of soluble polysulfide intermediates in Li–S battery electrolytes, while the cell operates, has the potential to have a widespread impact across the field, for example, by enabling the influence of a variety of polysulfide mitigation strategies to be assessed and optimized, including in this work the LiNO_3_ additive.

## 1 Introduction

As the world attempts to diversify its energy provision toward sustainable and renewable technologies, there is an increasing societal acceptance of alternate means of energy generation and storage. Next-generation lithium sulfur (Li–S) batteries are set to play a key role in this future energy landscape as they have theoretical gravimetric specific capacities an order of magnitude above current lithium ion (Li-ion) batteries (1672 mA h g^–1^), and theoretical energy densities over 5 times higher (2567 Wh kg^–1^).^1^ They are also safer and more sustainable, using sulfur for the cathode, the 16th most naturally abundant element in the Earth’s crust and the 4th most extracted, in lieu of toxic cobalt, nickel, and manganese transition metals which are often sourced unethically.^2−4^ However, Li–S batteries are hindered by the polysulfide (PS) shuttle effect—one of the most critical issues to solve in lithium sulfur batteries to enable their wider proliferation and commercialization.^5^ PS shuttling occurs when high-order soluble lithium PSs, Li_2_S_*x*_ (6 ≤ *x* ≤ 8), are generated at the cathode, diffuse toward the lithium metal anode, and undergo parasitic reactions to form lower-order PSs, Li_2_S_*x*_ (2 ≤ *x* ≤ 4). This leads to severe active material loss, degradation of the anode solid electrolyte interphase (SEI), the acceleration of dendrite formation, and subsequently rapid cell failure.^6^

The significant detriment of the PS shuttle effect on Li–S performance means that it has been a focus of the majority of research output on Li–S batteries (Figure S1). In spite of this, comparatively little work has gone into methods of characterizing the extent of PS shuttling, and thus assessing the efficacy of measures taken specifically to mitigate against it. Currently, researchers are largely reliant on improvements to Columbic efficiency and cycle life to prove the efficacy of shuttle mitigation techniques.^7−16^ However, these data only provide a consequential understanding of shuttling via overall cell performance which, considering the complexity of Li–S battery chemistry, can be impacted by numerous factors beyond PS shuttling. Full cycle life assessment is also incredibly time-consuming, typically requiring hundreds of cycles (corresponding to hundreds of days at the typical rate of C/10).

Several alternative methods to characterize PS shuttling have been outlined in the literature. For example, one incredibly simple method involves visibly observing the diffusion of deep brown PS from one H-Cell to another across a separator or interlayer. The extent of diffusion is representative of the material’s efficacy in inhibiting shuttle.^15,16^ This method is fast, cheap, and easy but is purely a study of diffusion and thus lacks any sort of electrochemical aspect. Nothing can be gleaned about the effect the fluctuating PS concentration throughout cycling may have, nor can the method be used to assess the impact within the whole cell. Additionally, monitoring the open-circuit potential of the cell and taking the rate of self-discharge as an analogue for the PS shuttle is both quick and extremely facile, but provides no spatial and limited electrochemical information, rendering it still difficult to draw a definitive conclusion on how or why a shuttle mitigation method works to improve performance.^17^ It also requires cycling to pause and hence cannot measure the impacts of charge/discharge dynamically (i.e., it is not an *operando* technique). Other methods of directly visualizing PS spatial distribution include neutron depth profiling (NDP) of the ^6^Li isotope, providing a high-resolution, nondestructive, spatially resolved, and *operando* measurement of lithium concentration (and thus the concentration of intermediate lithium PSs).^18^ This is however restricted to one-dimension (1D) and a limited depth of ∼21 μm (omitting a large proportion of electrolyte), while also requiring complex and expensive beamline instrumentation and a high degree of expertise. ^1^H magnetic resonance imaging (MRI) can also yield nondestructive *operando* visualization of PSs throughout cycling, wherein the presence of dissolved PS enhances the MRI signal and thus indirectly indicates the PS spatial distribution.^19^ However, this technique indirectly detects PS through enhancement of a certain ^1^H signal and thus may omit certain PS chain lengths, while also being a difficult procedure requiring highly specialist equipment. Quantitative assessment of PS concentration is possible using optical resonance combs, as demonstrated in work from Liu et al.^20^ Using tilted fiber Bragg grating (TFBG) sensors placed in the electrolyte of an operando Li–S cell, the refractive index could be correlated with the bulk PS concentration within the electrolyte. Further, temperature and strain could be measured with the probes, which enabled the authors to demonstrate the strong links between cycling performance and PS dissolution and precipitation phase transitions. The technique is very robust in that it allows simultaneous monitoring of several properties, alongside quantitative electrolyte PS concentration. However, the probe can only provide bulk assessment of the electrolyte PS concentration, eliminating any possibility of assessing PS concentration gradients or spatial distribution and thus the extent of shuttle or kinetics of PS within the electrolyte. Additionally, the method is very complex, requiring extensive and difficult data analysis, which the authors themselves state limits the applicability of the technique. The probe itself is also expensive and bulky, further limiting the environments and Li–S systems in which this valuable technique can be easily employed. A facile method to visualize PSs with high spatial and temporal resolution in two-dimensions (2D), allowing rapid acquisition and using widely available equipment, would be of significant utility to the Li–S research community.

Optical microscopy, a tool that is widely available and often simple to utilize, has previously been used to study the PS-induced color change of the Li–S electrolyte, similarly to the above-mentioned H-cell diffusion technique, except optical microscopy can be used during *operando* cycling.^21^ This allows nondestructive and fast *operando* estimations of electrolyte PS distribution through correlating changes in image color or contrast with PS concentration, and the resulting dendrite formation arising from the shuttle can be visualized. However, as optical microscopy is limited to the wavelength of visible light, it offers relatively low-resolution imaging and tracking ability. The method is also limited to only observing very low concentrations of PS, as the separator will quickly reach color saturation. Fully transparent and colorless electrolytes must also be employed as to ensure any color change arises purely from PS proliferation. Further, the data produced by this method is at best qualitative, due to the complex makeup of the Li–S electrolyte making correlations of color change to PS distribution alone tenuous.

Optical fluorescence microscopy (OFM) provides a more selective solution for characterization and is used extensively in biological systems and materials studies such as tracking cell signaling molecules or monitoring catalytic activity.^22,23^ It offers the ease of use of optical microscopy but ensures selective imaging of only the fluorescent species within the sample; fluorescence intensity can then be directly and quantitatively correlated to the concentration of the species.^24^ OFM can achieve impressive temporal resolution of hundreds of frames per second, high spatial resolution down to hundreds of nanometers with ease, and is inherently nondestructive for *in situ* and *operando* studies.^25^ OFM also enables illumination of large excitation areas, enabling the rapid, facile, and highly specific tracking of relevant species across the entire electrolyte simultaneously.

OFM relies on the excitation of the sample through bombardment with visible and ultraviolet (UV)-light wavelengths and subsequent detection of weak emitted light of a different wavelength, which is separated from incident light through spectral emission filters fitted to the microscope.

OFM has previously been applied to battery materials. Padilla et al. attempted to selectively study the movement of lithium ions through a microfluidic channel saturated with a Li-ion-selective fluorescent dye through OFM.^26^ Solid LiCl was added to the top of the channel, and as it slowly dissolved, the movement of the released Li ions through the channel served as an analogue for the diffusion of Li ions through the battery electrolyte. As the Li ions diffused and their concentration increased, the fluorescence signal increased along the channel. From this, a model could be established to determine the diffusion coefficients of Li ions in different electrolytes. However, the setup undergoes no electrochemical control, observing only a simple one-way diffusion of lithium ions, and as such is unrepresentative of a real battery. Further, no attempt is made to quantify the localized Li-ion concentration from fluorescence intensity.

An OFM-based technique will have some advantages and disadvantages versus other characterization techniques. For example, optical microscopy is an easy and very robust method of characterizing phenomena which create significant physical changes. For Li–S, the full-color imaging possible with optical microscopes enables rich analysis of lithium metal dendrite formation - including limited three-dimensional (3D) imaging using depth profiling.^27,28^ Furthermore, significant releases of PS into the electrolyte will cause discoloration, which can be taken as a proxy for PS distribution.^21^ This is purely qualitative, however, and as with the dendrite formation, the limited information gleaned makes it difficult to deconvolute and tie these observations to specific processes occurring within the cell. In situ Raman spectroscopy, Raman mapping, and UV–visible spectroscopy (UV–vis) on the other hand provide much more information about the chemical and electronic structure of materials. They are used in Li–S for quantifying the electrolyte composition, giving insight into the concentration of PS and approximate chain length distribution.^29,30^ However, as they generally use point spectra, they lack any spatial resolution when employed for operando study, limiting the capability of characterizing PS shuttle and near-electrode phenomena. They also lack the ease of optical microscopy.

OFM offers many of the benefits offered by both optical and spectroscopic techniques. Using a PS-sensitive fluorescent dye, an OFM technique could correlate spatially resolved fluorescence intensities to a quantitative assessment of PS concentration across the electrolyte, while also visualizing dendrites as 2D silhouettes.^31^ This would offer a much higher sensitivity and specificity for electrolyte PS than optical microscopy and enable dendrite growth to be linked to changes in the electrolyte, although the imaging of the dendrites themselves would be much more limited. Furthermore, it would possess the ability to quantify the electrolyte PS concentration offered by in situ Raman, Raman mapping, and UV–vis, but in a spatially resolved manner and with the ease and speed of optical microscopy.

Qi et al. employed cadmium sulfide quantum dots as an additive to a multiwalled carbon nanotube cathode. The quantum dots were intended to adsorb and immobilize PSs, while also being naturally fluorescent–with the fluorescence response increasing upon binding. Through *in situ* fluorescence spectroscopy and confocal fluorescence imaging, they observed an increase in the quantum dot fluorescence response during discharge and concluded that this indicated successful adsorption of the PSs by the quantum dots and thus prevention of the shuttle. This is a very interesting application of fluorescence-based characterization of lithium sulfur batteries, and is a facile way of proving their successful trapping of the PSs and suppression of the polysulfide shuttle effect. However, while cadmium sulfide quantum dots are effective, they are also a dangerous and unsustainable material unlikely to be viable for broader usage in Li–S batteries. Further, the technique is only indirectly capable of characterizing the PS shuttle, through the increase in binding to the specific quantum dot additive. As a result, it has limited application in verifying the efficacy of other polysulfide mitigation techniques, such as alternative cathode additives or morphologies, or changes to the electrolyte. The quantity of PS not absorbed by the quantum dots, and thus the true extent of shuttle mitigation provided, is also unknown. Additionally, the method focuses solely on the cathode, which provides limited opportunity for study of PS shuttle and dissolution compared to the study of the electrolyte and electrode interfaces.

In this work, we report the application of *operando* OFM to study Li–S battery electrolyte and electrode interfaces, enabling facile and potentially quantitative visualization of the proliferation and spatial distribution of dissolved PS concentration within the electrolyte throughout cycling. Through the development of a fluorescent tag that binds to PSs, brightness profiles observed throughout cycling are shown to reflect the expected fluctuations in PS distribution, providing direct visualization of the PS shuttle effect. This tool is then used to demonstrate the importance of the anode-protective LiNO_3_ additive, elucidated through the catastrophic extent of dendrite formation observed upon its removal, in spite of evidence of a consistent extent of shuttle. The ease of use of OFM allows facile application to evaluate shuttle mitigation techniques, such as new cathodes, electrolyte additives, and separators. This technique will help provide new research insights and speed up development significantly in the area of solid–liquid phase reactions.

## 2 Materials and Methods

As none of the components used in a typical Li–S battery are inherently fluorescent, a suitable fluorescent dye was required, which fluoresces only in the presence of the relevant species. When the fluorescent dye binds to the relevant molecule, a nonradiative excited-state decay pathway for relaxation is shut off, causing the emission of fluorescent light. The binding of the dye molecule can be tailored to be specific to a certain molecule, achieving a high degree of selectivity useful for complex electrolytes.

A variety of hydrogen polysulfide fluorescent dyes were found in the literature which could be adapted for use on lithium polysulfides.^31−53^ The chosen dye was first synthesized by Zhou et al. for use in tracking the metabolic cell signaling molecule H_2_S_2_ in zebrafish via confocal fluorescence imaging. Its broad linear range, ease of synthesis, fast equilibration time, and crucially its high degree of selectivity for PSs among other reactive species made it favorable for use in Li–S battery study via OFM. The dye was synthesized according to the procedure described in the work from Zhou et al. and outlined in Figure S2.^31,53^ A solution of 4-nitro-1,8-napthalic anhydride (214 mg, 1 mM, Sigma-Aldrich, 95% purity) dissolved in 20 mL of ethanol was prepared. Into this solution was added dropwise a mixed solution of butylamine (146.28 mg, 2 mM, Sigma-Aldrich, 99.5% purity) and triethylamine (200 μL, Fisher, 99.7% purity). The mixture was heated to reflux for 6 h, before solvent removal via a rotary evaporator, and purification with silica gel column chromatography using a dichloromethane (DCM, anhydrous, Sigma-Aldrich, ≥ 99.8% purity) and methanol (anhydrous, Sigma-Aldrich, 99.8% purity) (v/v, 15:1) eluent. This gave 2-butyl-6-nitro-1*H*-benzo[*de*]isoquinoline-1,3(2*H*)-dione (PS-Li_2_S_*x*_) as a brown solid (221.19 mg, 74.1% yield). ^1^H NMR and ^13^C NMR spectra are provided as Figures S3 and S4, respectively.

Standard solutions of PSs are produced according to the method employed by Dibden et al.^54^ The preparation of Li_2_S_*n*_ solutions was performed in an Ar-filled glovebox. Lithium sulfide (Li_2_S, Alfa Aesar, 99.9% purity) and elemental sulfur (dried, Sigma-Aldrich, 99.98% purity) were added to glass vials in stoichiometric ratios to give the nominal average chain lengths (*n* = 2, 4, 6, 8), as calculated by eq 1 for saturated solutions. These salt mixtures were then dissolved in 10 mL of a mixture of 1,3-dioxolane (DOL, Sigma-Aldrich, anhydrous, 99.8% purity) and 1,2-dimethoxyethane (DME, Sigma-Aldrich, anhydrous, 99.5% purity) in the volume ratio of 1:1 (DOL/DME). To aid the dissolution of the salts, the solutions were heated to 60 °C and stirred for 72 h, before cooling to room temperature and stirring for a further 48 h. After the 48 h period, the saturated solutions (Figure S5) were decanted from the remaining powder cake.
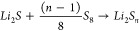
1

The performance of the PS-Li_2_S_*x*_ dye was assessed through several means. 100 μM Cetrimonium bromide (CTAB) was added while characterizing PS-Li_2_S_*x*_ in order to aid solubilization of PS-Li_2_S_*x*_ and raise reaction rates. CTAB was removed for the *operando* optical Li–S experiments, however, as the solubilization effect was not needed over the long time scale of the OFM experiments and to mitigate any chance of altered observations arising from the presence of an extraneous ionic additive. Spectro-fluoro-photometry was employed in order to characterize the fluorescent properties of PS-Li_2_S_*x*_, with the studies performed on a Shimadzu RF-6000 spectrofluorophotometer.

Charge/discharge cycling of Li–S cells containing PS-Li_2_S_*x*_ as an additive was conducted to ensure the inertness of the dye toward the standard performance and mechanism of operation. The nonoptical electrochemical cells were coin cells, prepared according to the common CR2032 format in a configuration standardized across the Faraday Insitution’s LiSTAR project.^55^ This consists first of the bottom coin cell case and a 0.5 mm spacer (PI-KEM), followed by the lithium metal anode (14 mm disc, Goodfellow), a separator (16 mm disc, Celgard 2400) wetted with 1 M LITFSI, 0.8 M LiNO_3_, and 1:1 (v/v) DOL/DME electrolyte (60 μL), a sulfur/carbon composite cathode (14 mm disc, NEI Nanomyte BE-70), and then another 0.5 mm spacer, a spring, and the top case (PI-KEM). The coin cell is then crimped to seal (MTI-140 coin cell crimper). When testing the impact of PS-Li_2_S_*x*_ on standard cell operation, 20 μM PS-Li_2_S_*x*_ was added to the electrolyte composition. Cells were cycled on a Biologic BCS-815, with 2 initial formation cycles at a rate of C/20, followed by constant current (CC) cycling at C/10.

To conduct the *operando* OFM imaging experiments, a cell was built using the EL-CELL ECC-Opto-Std optical cell employing a side-by-side imaging procedure, as depicted in Figure S6. The cell was fitted with a commercial sulfur/carbon composite cathode (NEI Nanomyte BE-70–70% sulfur, 20% carbon, 10% PvDF binder) and a 120 μm lithium foil anode (Sigma-Aldrich) both cut into semicircles of 14 mm diameter and arranged atop a glass fiber separator (EL-CELL) separated with an approximately 3 mm gap. The separator was saturated with approximately 0.3 mL of electrolyte (1 M LiTFSI, 0.8 M LiNO_3_, and 20 μM PS-Li_2_S_*x*_ in 1:1 (v/v) DOL/DME), and the gap then focused on to represent the electrolyte. This replicates the standard Li–S cell design employed for the electrochemical study commonly seen throughout the literature and standardized across the LiSTAR project.^55^ Charge/discharge cycling of the cell was performed with a Gamry Interface1000 potentiostat, using a current density of 0.7 mA cm^–2^ with respect to the active lithium metal surface area. The operating procedure of the methodology is outlined in Figure S7. Fluorescence imaging is conducted of the Li–S electrolyte saturated with PS-Li_2_S_*x*_ during cycling, and the intensity of the emitted fluorescence is proportional to the concentration of PS.

The concentration of PS in the electrolyte of a Li–S separator was determined through gravimetric analysis with barium nitrate to form barium sulfate.^54^ After cycling, the PS-saturated glass fiber separator was extracted to a round-bottom flask within a glovebox and sealed to retain the argon atmosphere. Five mL of ammonium hydroxide (20–30%, Sigma-Aldrich) was added, which caused the PS to dissolve into the solution from the glass fiber. Then, 5 mL of hydrogen peroxide (30% (w/w) in H_2_O, Sigma-Aldrich) was added with stirring to convert sulfur ions to sulfate ions, turning the solution colorless, which was then heated to 40 °C for 1 h to remove the excess ammonia and hydrogen peroxide. The solution was then diluted with deionized water, and 37% hydrochloric acid (HCl, Sigma-Aldrich) was added dropwise until the solution was slightly acidic (pH < 7). An excess of barium nitrate (synthesized from barium carbonate (≥99%, Sigma-Aldrich) and nitric acid (HNO_3_, 70%, Sigma-Aldrich)) was added to form barium sulfate with the sulfate ions, and the solution was stirred for 1 h. The final mixture was poured into a preweighed filter paper and filtered under vacuum to extract the barium sulfate. The retentate was rinsed with deionized water and allowed to dry at 50 °C for 24 h and subsequently weighed.

All fluorescence imaging was performed on a ZEISS Axio Zoom.V16 microscope with a ZEISS Illuminator HXP 200 C fluorescent light source. Excitation and emission fluorescent light was filtered (ZEISS Filter Set 38 HE) and collected by an EXview HAD CCD II camera (Zeiss AxioCam 506 color). Image processing and analysis were performed with the ZEISS ZEN 3.6 pro software, and within MATLAB using custom scripts. UV/vis analysis was performed with a Shimadzu UV-2600. Optical microscopy confocal depth profiling was conducted using a Keyence VHX-7000 digital microscope.

## 3 Results and Discussion

### 3.1 Synthesis and Characterization of Probe PS-Li_2_S_*x*_

To observe the movement of soluble lithium PSs within the electrolyte throughout *operando* cycling, a lithium PS-sensitive fluorescent dye, 2-butyl-6-nitro-1*H*-benzo[*de*]isoquinoline-1,3(2*H*)-dione (PS-Li_2_S_*x*_), was first synthesized and characterized (see Section 2). This enabled spatial tracking of PSs via the localized fluorescent response induced and monitored through OFM. When the nitro group on the fluorescent PS-Li_2_S_*x*_ molecule is reduced by the lithium PSs it becomes fluorescently active.

UV–vis absorption analysis evidences the PS-Li_2_S_*x*_ as a successful indicator of lithium PSs (Figure S8). Compared to the 4-nitro-1,8-naphthalic anhydride precursor, PS-Li_2_S_*x*_ has a slightly upshifted major peak, to 348 nm from 340 nm, arising from the change in structure, although the shape of the curve remains unchanged. In the presence of 100 μM Li_2_S_4_, the spectrum gains a shoulder peak below 320 nm, arising from a significant PS-induced change in structure.

**Figure 1:**
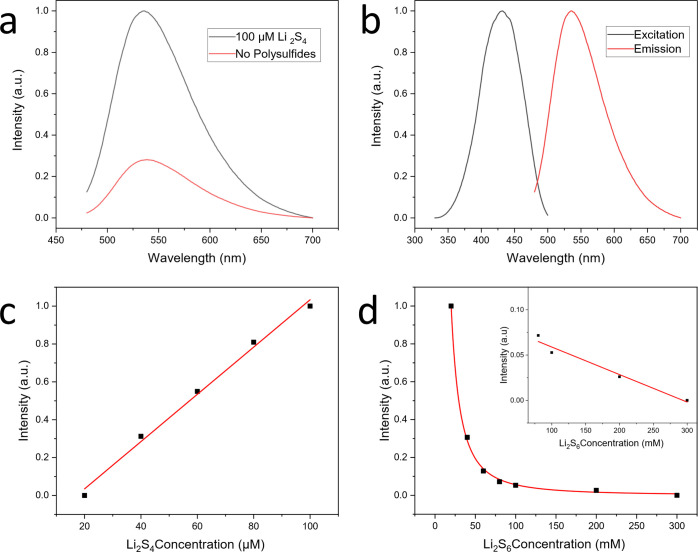
Spectrofluorophotometry characterization of PS-Li_2_S_*x*_ with Li_2_S_4_ PS addition. (a) Excitation and emission spectra for 100 μM PS-Li_2_S_*x*_ and 100 μM CTAB with 100 μM Li_2_S_4_ in methanol; λ_ex_ = 430 nm, λ_em_ = 535 nm. (b) Emission spectra collected at λ_ex_ = 430 nm for 100 μM PS-Li_2_S_*x*_ and 100 μM CTAB with and without the addition of 100 μM Li_2_S_4_. (c) Fluorescence intensity of 100 μM PS-Li_2_S_*x*_ and 100 μM CTAB in methanol on the addition of 20–100 μM Li_2_S_4_, with best-fit line. (d) Fluorescence intensity of 100 μM PS-Li_2_S_*x*_ in 1 M LiTFSI and 1:1 (v/v) DOL/DME on addition of 20–300 mM Li_2_S_6_ with best-fit curve. Inset: Linear quenching effect demonstrated between 80 and 300 μM with best-fit line.

Two areas of peak fluorescence intensity were observed through spectrofluorophotometry for a solution of 100 μM PS-Li_2_S_*x*_, 100 μM PSs, and 100 μM CTAB, representative of an excitation wavelength (λ_ex_) at 430 nm and an emission wavelength (λ_em_) at 535 nm (Figure 1). The fluorescence intensity at λ_em_ notably correlates linearly with Li_2_S_4_ concentration from 10 to 100 μM. This is the linear range of the dye and is in agreement with the linear range presented in the literature. The λ_ex_ and λ_em_ of PS-Li_2_S_*x*_ represent a large Stokes shift of 105 nm, and so there is a minimal chance of error in fluorescence detection from phenomena such as an overlap of emission/excitation wavelengths.

For PS-Li_2_S_*x*_ to be applicable to an *operando* Li–S system, it needed to selectively react with only the lithium PSs within the electrolyte and be entirely inert to the mechanism of operation. Zhou et al., in using PS-Li_2_S_*x*_ for tracking the H_2_S_2_ signaling molecule in complex biological environments, reported it to have high selectivity for PS among reactive oxygen, reactive nitrogen, and reactive sulfur species.^31^ The fluorescence response of PS-Li_2_S_*x*_ was first probed when exposed separately to sulfur, a 0.1 M solution of the Li_2_S_4_ intermediate PS, and the pure electrolyte (1 M LiTFSI and 0.8 M LiNO_3_ in 1:1 (v/v) DOL/DME). No notable fluorescence response was produced by either sulfur or the electrolyte, regardless of the addition of PS-Li_2_S_*x*_, while the PSs were successfully found only to fluoresce in the presence of PS-Li_2_S_*x*_ (Figure S9).

During the OFM experiments, while at PS concentrations between 0.1 and 100 μM, the linear range of PS-Li_2_S_*x*_ (Figure 1c) can be exploited to extract the PS concentration measured at each pixel from fluorescence intensity, and so gain a quantitative assessment of the spatial distribution of PS. However, as noted in the literature, the operating concentration is generally far above this range in standard Li–S cells without polysulfide shuttle mitigation methods applied, being in the range of hundreds of mM.^20^ As seen in Figure 1d, outside of this range a concentration quenching effect is instead experienced, where fluorescence intensity decreases with increasing PS concentration, with the correlation even becoming linear at the concentrations expected in typical Li–S cells (>80 mM).^56^ The analysis at these concentrations is qualitative but can be combined with *ex situ* analysis for quantification. To understand the impact of different PS chain lengths, stock concentrations of each PS expected throughout cycling (Li_2_S_2_, Li_2_S_4_, Li_2_S_6_, and Li_2_S_8_) were prepared in 20 μM PS-Li_2_S_*x*_, and a glass fiber separator was saturated with the solutions before they were imaged using OFM. It has been shown previously that when PS-Li_2_S_*x*_ was utilized with hydrogen PS, its high selectivity caused significant depletion in the fluorescence response with increasing hydrogen PS chain length, with a decrease in fluorescence intensity of 37% from H_2_S_2_ to H_2_S_3_, and 93% from H_2_S_2_ to H_2_S_4_.^31^ However, this was not reflected in the lithium PS experimental data, as all PS chain lengths were observed to fluoresce to a consistent intensity, suggesting a mechanism of fluorescence more dependent on the ionic lithium component of the polysulfides and that fluorescence intensity is purely representative of the total PS concentration (Figure S10a). As such, a calibration curve was prepared showing the modal fluorescence intensity at varying concentrations of Li_2_S_4_ between 10 and 100 μM (Figure S10b).

**Figure 2:**
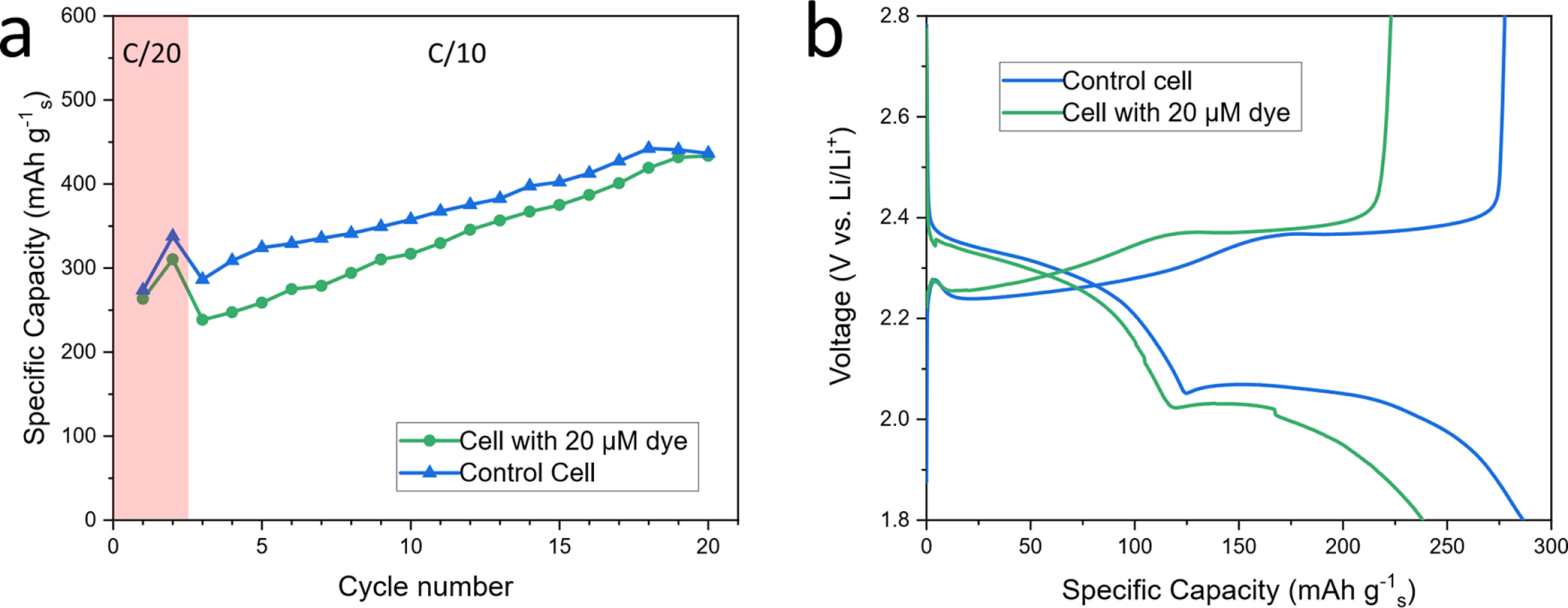
For a Li–S cell both with and without the addition of 20 μM PS-Li_2_S_*x*_, (a) specific discharge capacity (sulfur mass) against cycle number for 20 cycles, with 2 formation cycles at C/20 and a further 18 at C/10, and (b) cycling data showing specific capacity (sulfur mass) taken during the third cycles at C/10.

Next, to assess the impact of PS-Li_2_S_*x*_ on the charge–discharge behavior of Li–S batteries, coin cells were built consisting of a 14 mm diameter Li metal anode, a Celgard 2400 separator, a 14 mm (70:20 wt %) sulfur/carbon composite cathode, and 60 μL of electrolyte (1 M LiTFSI, and 0.8 M LiNO_3_ in 1:1 (v/v) DOL/DME) with and without 20 μM added PS-Li_2_S_*x*_. Very similar capacities and charge/discharge cycling curves were obtained upon the addition of PS-Li_2_S_*x*_ (Figure 2), showing that the fluorescent tag had little impact on the electrochemical response. The shape of the Li–S charge/discharge curves, as seen in Figure 2b, is indicative of the mechanism of operation, with the kinetics of the PSs indicated with two plateaus representing first the phase change of S_8_ dissolution, followed by Li_2_S/Li_2_S_2_ deposition. Variation in the expected shape of the curve would indicate a disruption in the traditionally understood mode of operation. Further, a significantly reduced capacity arising from the loss of active material would be observed if PS-Li_2_S_*x*_ binds irreversibly to, or otherwise denatures, the active PSs. As seen in Figure 2, the addition of 20 μM PS-Li_2_S_*x*_ caused very minor changes in either the shape of the charge/discharge curve, or in capacity, qualifying it for use in *operando* studies. The difference in capacities for the two cells is within the expected variance of the commercial standard NEI Nanomyte BE-70 cathodes used, which arises due to differences in cathode morphology and sulfur loading.^57^ Capacity increases beyond the third cycle due to the continual dissolution of sulfur increasing sulfur utilization.^57^

### 3.2 *Operando* Optical Fluorescence Microscopic Imaging of PS-Li_2_S_*x*_ in a Cycling Li–S Cell

A Li–S cell was created within a specialized *operando* optical microscopy cell (EL-CELL) depicted in Figure S6 employing an optical side-by-side cell imaging procedure. The components used were representative of a common Li–S coin cell.^55^

The cell was cycled for 7 complete charge/discharge cycles with a current density of 0.7 mA cm^–2^ with respect to the area of lithium (below the critical current density for dendrite formation of 1 mA cm^–2^).^58^ Light, at the excitation wavelength, was shone onto the electrolyte gap, while images were taken every minute of the fluorescence response across the separator at the emissive wavelength. The cycling curves produced from the *operando* optical cell again matched that expected for Li–S, despite high resistance from a substantial 3 mm electrolyte gap and small active surface area to electrode volume ratio. PS-Li_2_S_*x*_ is reported to have a full fluorescence activation time of 20 min. Due to the rate of cycling employed, where the average discharge/charge step took roughly 10 h, this activation time did not lead to inaccuracy on this time scale. The relevant cycling data and 1D averaged electrolyte gray values are available in the Supporting Information.

**Figure 3:**
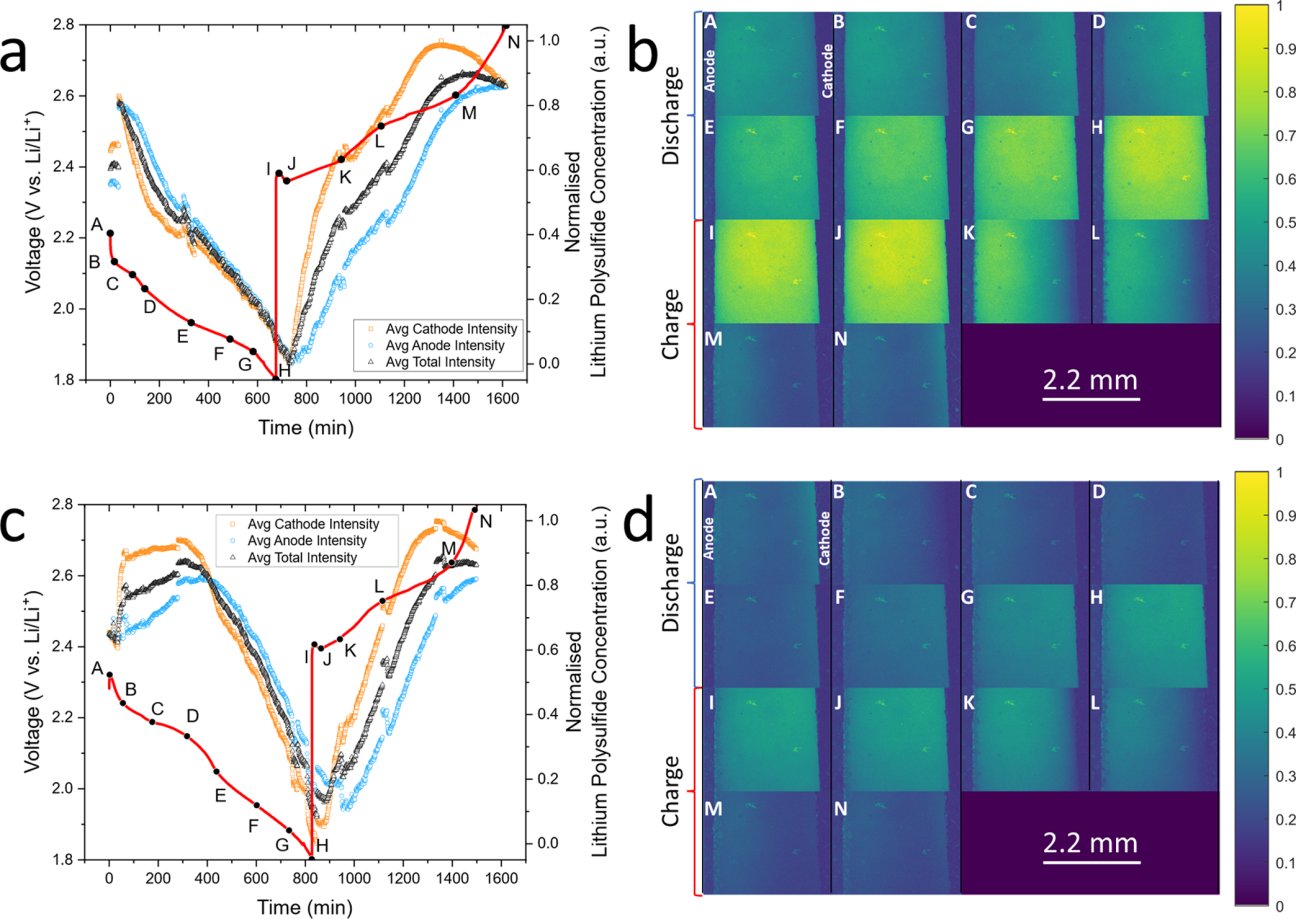
Cycling data (a, c) and optical fluorescence imaging (b, d) taken during operando study of the electrolyte of a standard Li–S cell, with 20 μM polysulfide-sensitive PS-Li_2_S_*x*_ fluorescent dye for the first cycle (a, b) and the second cycle (c, d).

The brightness across the imaged electrolyte was seen to vary throughout the charge–discharge cycling, resulting from the fluctuations in localized PS speciation and concentration in the electrolyte at various potentials. Figure 3 shows the fluorescence images taken at mechanistically significant potentials from the first two cycles. The initial image, taken at the open-circuit potential of 2.21 V (Figure 3b.A), shows a degree of fluorescence rather than being completely inactive, reflecting that even before the application of current, some of the solid S_8_ within the cathode had dissolved into the electrolyte as Li_2_S_8_ in a form of self-discharge, reaching an equilibrium concentration. The dissolution of S_8_ into the electrolyte as Li_2_S_8_ at OCV is an expected phenomenon and is reflected in UV–vis studies.^59^

The behavior of PS-Li_2_S_*x*_ in this experiment is caused by the concentration of the electrolyte being outside of the linear range of the fluorophore (0.1 to 100 μM). The concentration of PS was measured in the separator of a cell charged to the voltage of expected maximum dissolution (2.7 V, as observed in work from Liu et al.) using barium sulfate gravimetric analysis and was found to be 281 ± 8 mM.^20,54^ As mentioned previously, at concentrations beyond the linear range, fluorescence concentration quenching occurs. This results in a clear inverse correlation between expected PS concentration, and fluorescence intensity (Figure 1d).^56^ In the study of a high PS concentration electrolyte, fluctuations in fluorescence intensity corresponding with the inverse expected fluctuation in PS concentration (from Li_2_S_2_/Li_2_S and S_8_ dissolution and deposition) can be attributed to fluorescence concentration quenching. This provides a useful, though qualitative, assessment of the spatial proliferation of PS in the electrolyte of the cycling Li–S cell. The overall bulk electrolyte concentration trends reflect those found with more complex methods in existing literature.^20^ In the ideal Li–S cell, however, to maximize sulfur utilization and minimize shuttle, the PS concentration within the electrolyte will be as low as can be achieved, ideally below the 100 μM linear range. Within this range, the fluorescence will correlate linearly with PS concentration, allowing for a more precise and quantitative assessment of PS electrolyte phenomena (Figure 1d).

As the discharge begins, the first discharge plateau is observed until 2.10 V (Figure 3b.C). At this potential, the S_8_ produced via self-discharge begins to be deposited as Li_2_S and an increased fluorescence, and thus a decreased PS concentration, is observed at the cathode. Darkening is conversely observed at the anode, correlating to increasing PS concentration, due to diffusion of solubilized PS to the anode down the concentration gradient.

During further discharge, below 2.10 V, brightness at the cathode increases as PS are driven further toward the slow process of Li_2_S/Li_2_S_2_ phase change precipitation, decreasing PS concentration (Figure 3b.C–H). Also visible during this period is a sweeping brightness from the cathode toward the anode representing a loss of PS from the anode, both through parasitic reaction with the anode depleting the SEI and diffusion of PS toward the cathode down the concentration gradient arising from Li_2_S/Li_2_S_2_ precipitation.

Little change appeared as charge began, and solid Li_2_S/Li_2_S_2_ dissolution was induced, other than for the genesis of dendrite formation near the anode (Figure 3b.I,J). As the OFM method is still optical imaging (though of fluorescent areas of which only the liquid state PS can induce a fluorescence response) the solid electrodes appear as dark silhouettes. Dendrite formation can thus be seen in the fluorescence images through the appearance of growths in the silhouette of the once flat lithium metal anode. Further into charge, beyond 2.36 V, the brightness at both electrodes decreases and significant dendrite formation is observed (Figure 3b.K,L). At the cathode, the decreased fluorescence is due to a gradual gain in PS concentration from Li_2_S_2_/Li_2_S dissolution. At the anode, the PS concentration increase arises from the PS shuttle effect, which is directly visualized as the sweeping of darkness (representing PS) from the cathode to the anode (Figure 3b.K–N). This is direct observation of the shuttle effect, arising from the diffusion of high-order PS to the anode, and subsequent parasitic reactions between the PS and SEI which form lower-order PS while also degrading the SEI.

At around 2.6 V, the PS concentration plateaus at the anode and decreases at the cathode. This is due to S_8_ deposition depleting the concentration of solubilized PS at the cathode, and subsequently flattening the concentration gradient and halting the PS shuttle to the anode.

Significant dendrite formation was observed during the lithium plating of the charge step, despite staying below the critical current density for dendrite formation of 1 mA cm^–2^ and is clearly the result of intense SEI degradation. As noted above, high concentrations of PSs were consistently observed at the anode due to a harsh extent of the PS shuttle, leading to stripping away of the anode protection layer from parasitic reactions.

The average fluorescence intensity is also taken across the entire electrolyte and for the isolated cathode and anode sides (Figure 3a). Minimal fluorescence intensity, and thus maximal PS concentration, is found at the end of the first discharge plateau and at the end of charge. The highest intensity is at the end of discharge, featuring the lowest concentration of PS due to Li_2_S_2_/Li_2_S precipitation. The anode is notably brighter/harbors a lower PS concentration than the cathode at the end of charge/start of discharge (Figure 3b.A,3d.B). The anode then “overtakes” the cathode concentration at the end of the first discharge plateau, where mass deposition of Li_2_S_2_/Li_2_S begins at the cathode. The anode gains PSs from diffusion, which react parasitically with the SEI. At the first charge plateau, the concentration of PSs at the cathode interface again increases beyond the anode due to the dissolution of Li_2_S_2_/Li_2_S increasing concentration. An increase in average cathode fluorescence intensity can be seen at the end of charge around the second charge plateau, representative of S_8_ precipitation rapidly lowering the PS concentration at the cathode interface.

The fluorescence changes reflect the expected trends in spatial lithium PS concentration, evidencing the method as appropriate for a facile *operando* study of electrolyte PS distribution. The most pertinent observation is that of direct PS shuttling (Figure 3b.J–M,d.J–M). It is notable that in spite of the expectation of a return shuttle of an equal amount of low-order PS from the anode back to the cathode, the PS concentration remained consistently high at the anode. This suggests that the shuttle effect may be more detrimental than expected, as the consistently high concentration of PSs at the anode will continue to contribute to SEI degradation and increase the active material loss.

The second cycle shows the expected trends in PS movement throughout, similar to the first cycle described above. Unlike the first cycle, however, it begins with a notably higher PS concentration at the cathode than at the anode, with the PS concentration at the cathode dropping below the anode at the end of the first discharge plateau (2.03 V). This is replicated for all future cycles (Figure S11). This is a result of the first cycle beginning after a considerable rest period, during which an equilibrium concentration of PS was reached throughout the electrolyte. All subsequent cycles begin immediately after the previous charge step, during which solid Li_2_S_2_/Li_2_S dissolution increases the PS concentration at the cathode above that of the anode. Cathode PS concentration then drops below the anode as discharge induces Li_2_S_2_/Li_2_S deposition.

The second cycle is also darker overall than the first cycle. This is due to the increased contact time between the electrolyte and the cathode, granting a higher overall concentration of PS within the electrolyte and increasing the fluorescence quenching effect. The increasing concentration of PS within the electrolyte is representative of capacity fade as the electrolyte active material is inaccessible to the cathode. Seven complete charge/discharge cycles were completed during this experiment for this cell, with the same phenomena observed throughout cycling as detailed for the first and second cycles above (Figure S11). Peak fluorescence intensity decreases until the third cycle, at which point it fluctuates around the same level (around 47% of the initial peak fluorescence intensity), correlating with the cell reaching a more stable capacity. Continual dissolution of PS into the electrolyte throughout cycling may be expected, arising from the continued contact between the electrolyte and active sulfur material. It is likely that this plateau at the third cycle is due to the electrolyte reaching a saturation point for dissolved PS, also stabilizing the corresponding capacity fade from active material loss.

### 3.3 *Operando* Optical Fluorescence Microscopic Imaging of PS-Li_2_S_*x*_ in a Cycling Li–S Cell without LiNO_3_

**Figure 4:**
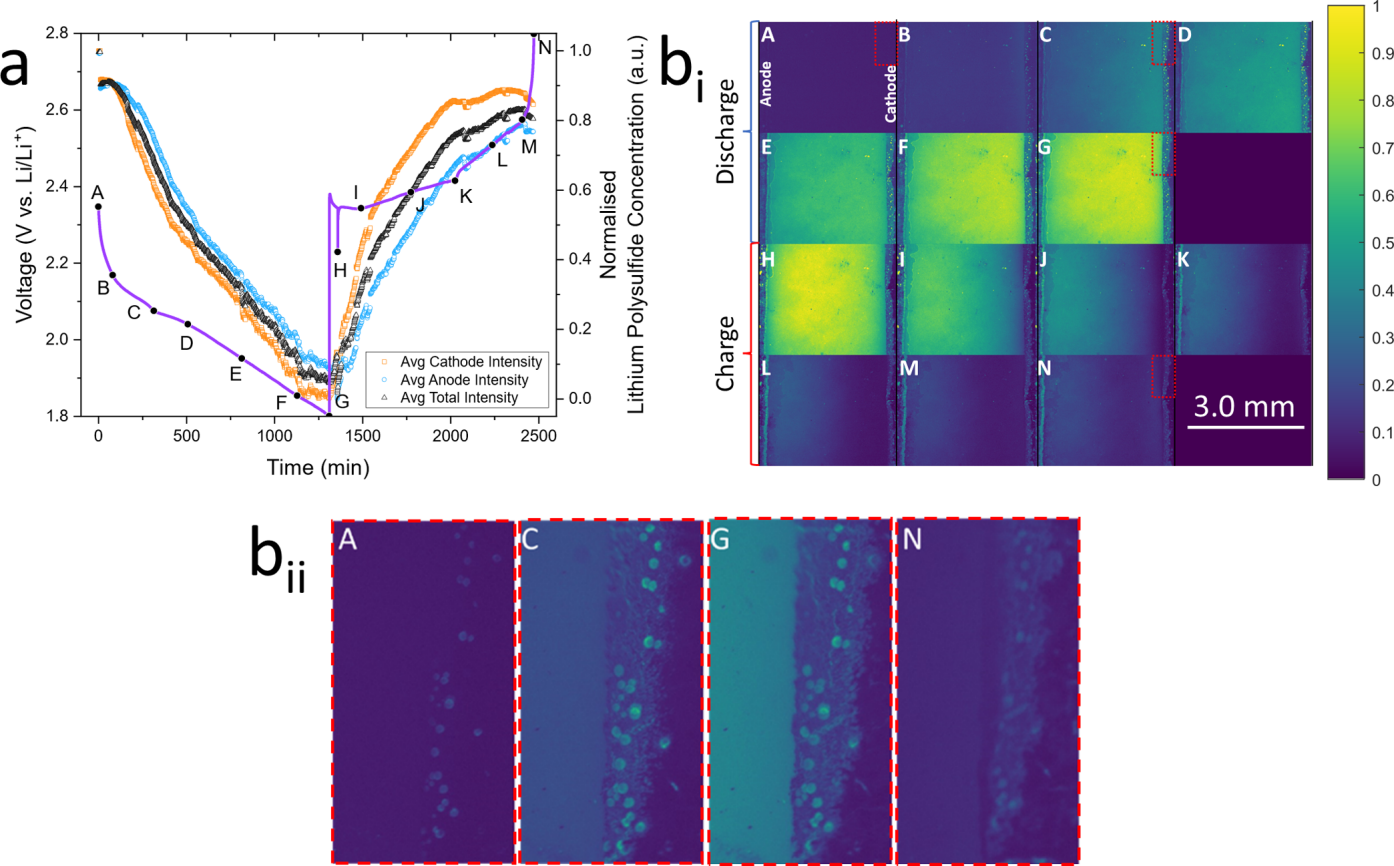
Cycling data (a) and optical fluorescence images (b_i_, b_ii_) taken during operando study of the electrolyte of a standard Li–S cell, omitting the SEI protective LiNO_3_ additive, with 20 μM polysulfide-sensitive PS-Li_2_S_*x*_ fluorescent dye for the first cycle. (b_ii_) Enlarged fluorescence images of pores within the cathode and nearby electrolyte during the first cycle at (A) open-circuit potential, (C) first discharge plateau, (G) end of discharge, and (N) end of charge.

To demonstrate the applicability of OFM to study the impact of electrolyte additives on lithium PS shuttling and other common cell processes, a Li–S cell omitting the common additive LiNO_3_ from the electrolyte was also visualized using the fluorescence method. LiNO_3_ is added as a sacrificial additive to stabilize the SEI against parasitic reactions with PSs and keep the lithium metal anode protected. When PSs reach the anode, a high degree of gas evolution from SEI degradation was observed from air bubbles on the metal surface (Figure 4b_i_.C–N), leading to increased resistance and suggesting increased degradation relative to the prior LiNO_3_-containing cell. This is further evidenced by an increased level of dendrite formation, particularly during the second cycle (Figure S12b.G).

While qualitative comparisons can be made between these two experiments, due to both cells exploiting the fluorescence quenching effect, quantitative comparisons of PS concentration cannot be made between them. Despite this cell having the same composition as the previous experiment, the overall fluorescence intensity of the cell was 52% lower, meaning that the overall PS concentration was higher without LiNO_3_. This is due to the less saturated electrolyte accommodating increased dissolution of PS.

The shuttle effect can be seen to occur similarly both with and without LiNO_3_, but only without LiNO_3_ does dendrite formation occur so severely. Hence, these data demonstrate that OFM is an ideal platform for the characterization of a wide variety of other shuttle mitigation strategies, such as new additives, separators, or PS trapping cathode morphologies, where the spread of fluorescence will be visibly limited as the PSs are trapped by the separator or cathode.

Throughout the 11 complete charge/discharge cycles completed for this cell, a different phenomenon in the intensity profiles was noted than in the cell containing LiNO_3_ (Figure S13). Here, the peak fluorescence intensity continually decreases throughout cycling, correlated with a consistent significant capacity fade. This is due to the lack of SEI forming LiNO_3_ additive allowing continual parasitic reaction between PS and the lithium metal anode, leading to active material loss through the destruction of PS in the electrolyte and subsequently reduced electrolyte PS concentration.^60^ Another phenomenon observed in this study are “glowing spots” within the cathode, which represent pores containing an increased fluorescence intensity compared to the bulk electrolyte or at the cathode surface (Figure 4b_ii_). This arises from low concentrations of solubilized PSs trapped within pores at the visible top surface of the electrodes. The prominence of these pores can be seen via optical microscopy confocal depth profiling (Figure S14), with diameters of around 10–20 μm, and depth of approximately 10 μm. It is notable that at the first discharge plateau of the first cycle, where S_8_ begins its first dissolution, the PS concentration within these pores is considerably lower than in the surrounding electrolyte (Figure 4b.C). While at the end of discharge, an equilibrium low PS concentration is eventually reached with the electrolyte (Figure 4b.G), at the end of charge, the pores continue to contain a lower PS concentration than the electrolyte due to the enhanced redeposition of S_8_ at the end of charge (Figure 4b.N). These phenomena suggest that the observed pores cannot support high concentrations of dissolved PS. They perhaps instead contain regions of deposition behavior that are kinetically enhanced above the surrounding cathode regions. Further, the movement of fluorescence in and out from these pores throughout cycling can be correlated with both PS deposition and dissolution kinetics at these isolated surface-active areas, and with the rates of PS mass transport within the porous cathode morphology.

## 4 Conclusions

In this work, *operando* optical fluorescence microscopy has been demonstrated to be a powerful and cost-effective tool for quantitative study of the dynamics of lithium polysulfides within Li–S batteries.

The polysulfide-sensitive fluorescent dye PS-Li_2_S_*x*_ was synthesized and proven to fluoresce selectively in the presence of the polysulfide ions within a Li–S cell, while remaining inert with regard to the mechanism of standard cell operation. PS-Li_2_S_*x*_ was added to the electrolyte of a specialized Li–S optical cell, enabling potentially quantitative imaging of the spatial distribution of polysulfides within the electrolyte during *operando* cycling. Most notably, the polysulfide shuttle effect was directly observed, as was its impact on cell health through SEI degradation, leading to excessive dendrite formation. Additionally, the efficacy of the LiNO_3_ electrolyte additive in protecting the SEI from parasitic polysulfide reactions and reducing dendrite formation from polysulfide shuttling was observed.

This technique has therefore been demonstrated to be a highly promising tool for Li–S research, uniquely enabling rapid and facile characterization of PS shuttle mitigation techniques, such as new electrolyte additives, electrolyte compositions, cathode morphologies, and separators. The technique can also aid studies of the kinetics and mass transport processes within Li–S batteries and beyond. Most importantly, the relatively low cost of the experimental tools utilized, compared to available alternatives, combined with the facile experimental procedure, will allow this technique to be widely adopted for Li–S battery characterization across academia and industry.

## Supporting Information Available

Review of the relevance of polysulfide shuttle study in Li-S literature; synthesis procedure of PS-Li_2_S*_x_*; ^1^H NMR and ^13^C NMR of PS-Li_2_S*_x_*; stock solutions of PS in DOL/DME; schematic illustrations of the optical cell setup and the test principle of the technique; UV–vis of PS-Li_2_S*x* with and without PS; example images of PS-Li_2_S*_x_* selectivity; calibration curve for PS-Li_2_S*_x_* fluorescence intensity against PS concentration; cycling data and averaged regional fluorescence intensity for all cycles of the experiments; cycling data against key optical fluorescence microscopy images for the second cycle of the experiment; and optical microscopy confocal depth profile of the surface pores present on the NEI cathode (PDF)
