# Supplementary material for: Illuminating Polysulfide Distribution in Lithium Sulfur Batteries; Tracking Polysulfide Shuttle Using Operando Optical Fluorescence Microscopy
Source: ACS Appl Mater Interfaces. 2024 Apr 10;16(16):20329–40. doi: 10.1021/acsami.3c14612 (PMC11056927; doi:10.1021/acsami.3c14612)
Supplement: Supplementary file 1 — am3c14612_si_001.pdf [file am3c14612_si_001.pdf]

Supporting Information for:

Illuminating polysulfide distribution in lithium sulfur batteries; tracking polysulfide shuttle using *operando* optical fluorescence microscopy

Kofi Coke[1], Michael J. Johnson[1], James B. Robinson[1,2,3], Alexander J. E. Rettie[1,2,3], Thomas S. Miller[1,2]*, Paul R. Shearing[2,4]*

1. Electrochemical Innovation Lab, Department of Chemical Engineering, University College London, Torrington Place, London, WC1E 7JE, U.K.
2. The Faraday Institution, Quad One, Becquerel Avenue, Harwell Campus, Didcot, OX11 ORA, U.K.
3. Advanced Propulsion Lab, UCL East, University College London, London, E15 2JE, U.K.
4. Department of Engineering Science, University of Oxford, Parks Road, Oxford, OX1 3PJ, U.K.

* Email: t.miller@ucl.ac.uk; paul.shearing@eng.ox.ac.uk

Table of contents

S1: Literature review of the relevance of polysulfide shuttle study

S2: Synthesis procedure for PS-Li_2S_x

S3: 1H NMR of PS-Li_2S_x

S4: 13 C NMR of PS-Li_2S_x

S5: Stock solutions of polysulfides in DOL:DME

S6: Schematic of optical cell set-up

S7. Schematic illustration of the test principle of the optical fluorescence microscopy technique

S8: UV-Vis of PS-Li_2S_x with and without the addition of polysulfides

S9: Example images demonstrating PS-Li_2S_x fluorescence selectivity

S10: Calibration curve of PS-Li_2S_x fluorescence intensity against polysulfide concentration

S11: O*perando* optical fluorescence microscopy study of a standard Li-S cell showing cycling data and fluorescence intensity for all cycles

S12: *Operando* optical fluorescence microscopy study of an Li-S cell without the $LiNO_3$ additive

S13: O*perando* optical fluorescence microscopy study of an Li-S cell without the $LiNO_3$ additive, showing cycling data and fluorescence intensity for all cycles

S14: Optical microscopy confocal depth profiling of surface pores prominence within the NEI cathode

S1. Literature review of the relevance of polysulfide shuttle study

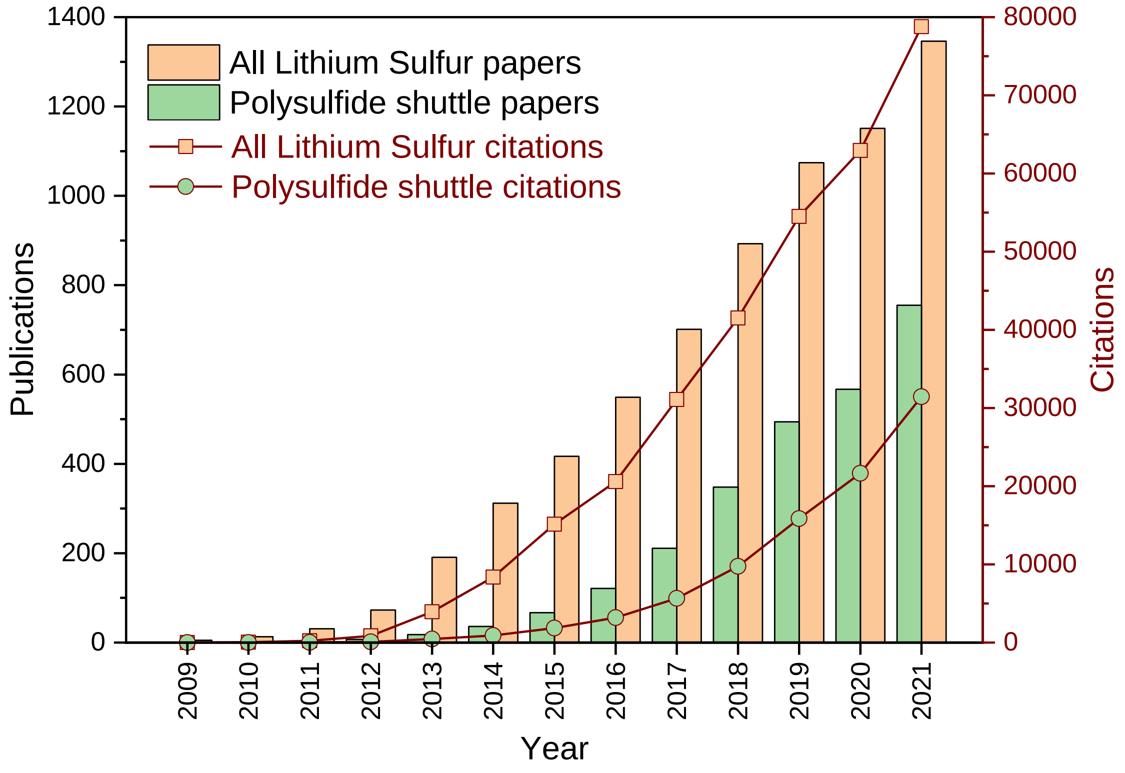

Figure S1: Number of publications relating to Li-S and the shuttle effect by year. “All lithium sulfur papers” data gathered through a Citation Report for title “lithium & sulfur” OR “lithium & sulphur” OR “Li-S” and topic “battery” on Web of Science, while “Polysulfide shuttle papers” data gathered similarly with a Citation Report using title “lithium & sulfur” OR “lithium & sulphur” OR “Li-S” and topic “battery” AND polysulfide shuttle.

S2. Synthesis procedure for PS-Li_2S_x

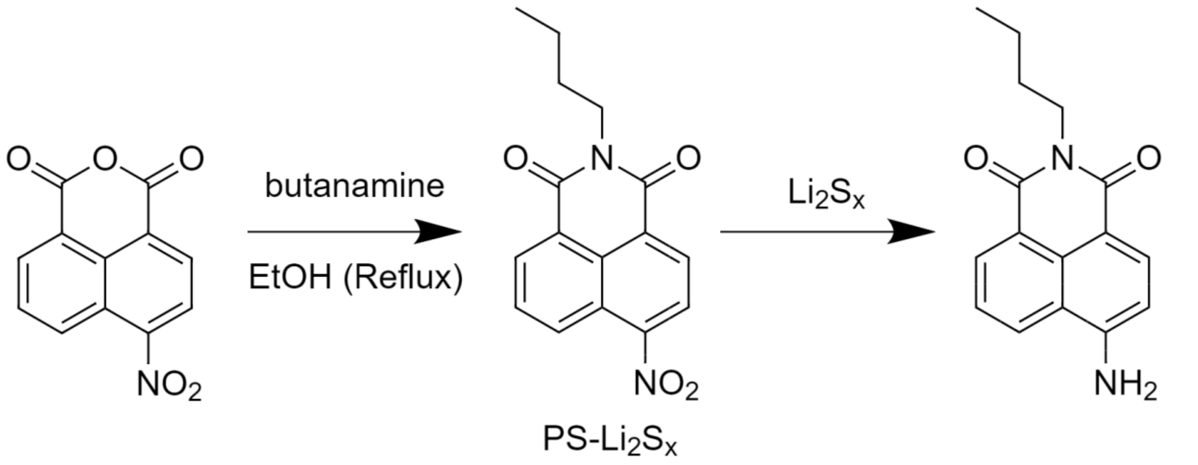

Figure S2: Proposed reaction scheme for the synthesis of the polysulfide sensitive fluorescent dye PS-Li_2S_x. The subsequent reaction of the dye with lithium polysulfides is also given.

S3. 1H NMR of PS-Li_2S_x

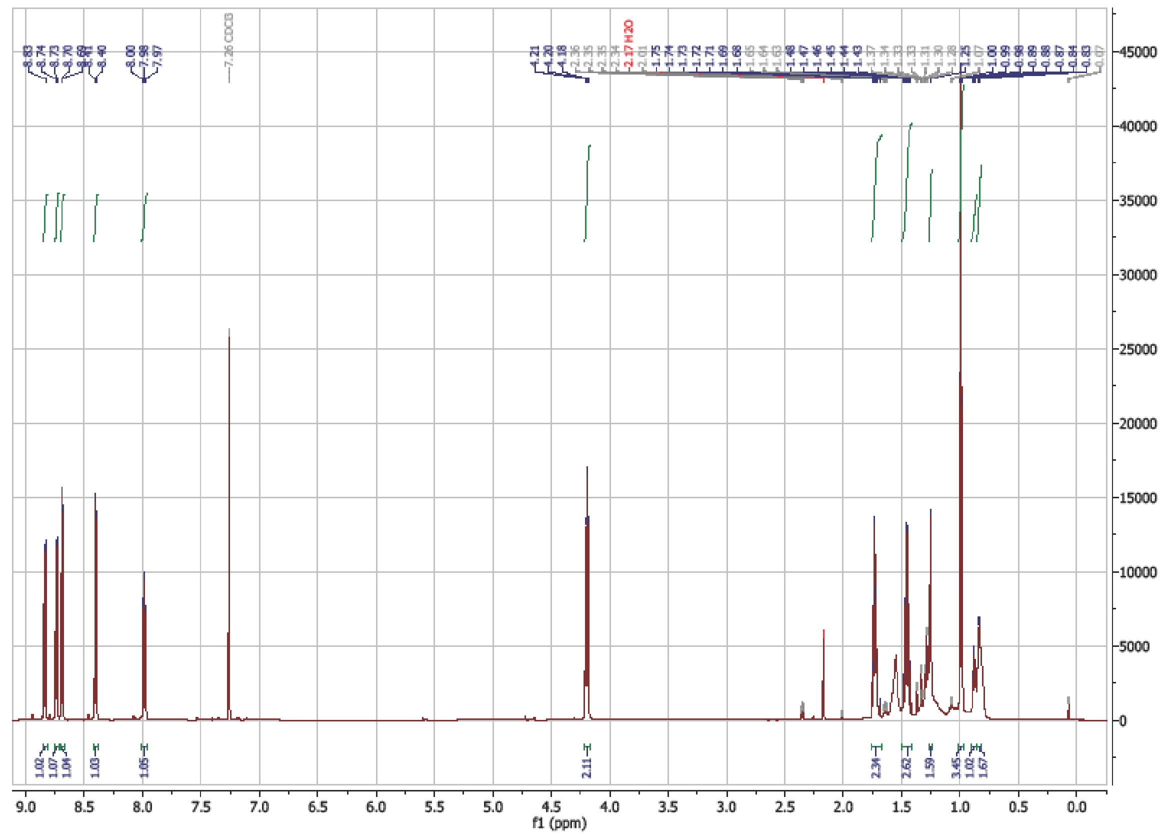

Figure S3: ^{1}H-NMR spectrum of dye (700 MHz, $CDCl_3$, 298K)

^{1}H NMR (700 MHz, $CDCl_3$, 298 K): δ 8.84 (d, J = 8.7 Hz, 1H), 8.74 (d, J = 7.2 Hz, 1H), 8.69 (d, J = 7.9 Hz, 1H), 8.40 (d, J = 7.9 Hz, 1H), 7.98 (t, J = 8.2 Hz, 1H), 4.19 (t, 7.6 Hz 2H), 1.76 – 1.67 (m, 2H), 1.46 (h, J = 7.4 Hz, 2H), 1.31 – 1.22 (m, 2H), 0.99 (t, J = 7.4 Hz, 3H), 0.90 – 0.79 (m, 4H).

S4. 13 C NMR of PS-Li_2S_x

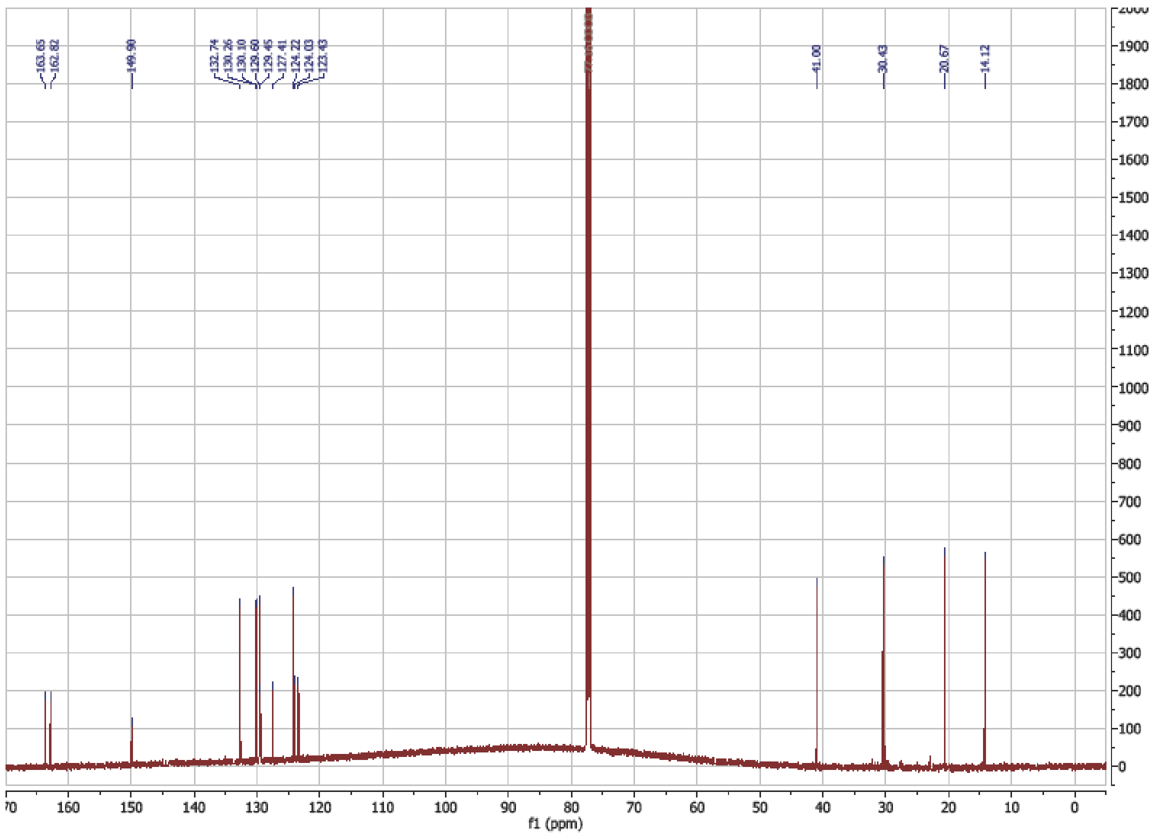

Figure S4: ^{13}C-NMR spectrum of dye (176 MHz, $CDCl_3$, 298K)

^{13}C NMR (176 MHz, $CDCl_3$, 298 K): δ 163.65, 162.82, 149.90, 132.74, 130.26, 130.10, 129.60, 129.45, 127.41, 124.22, 124.03, 123.43, 77.52, 77.34, 77.16, 41.00, 32.26, 30.43, 30.03, 23.02, 20.67, 14.45, 14.12.

S5. Stock solutions of polysulfides in DOL:DME

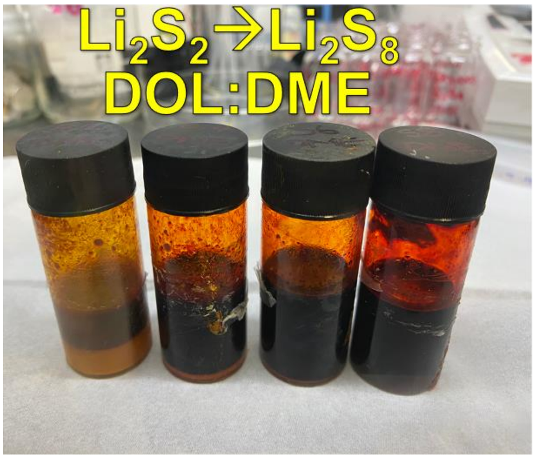

Figure S5: Stock solutions of saturated polysulfide solutions. (Left-Right) Li_2S_2, Li_2S_4, Li_2S_6, and Li_2S_8

S6. Schematic of optical cell set-up

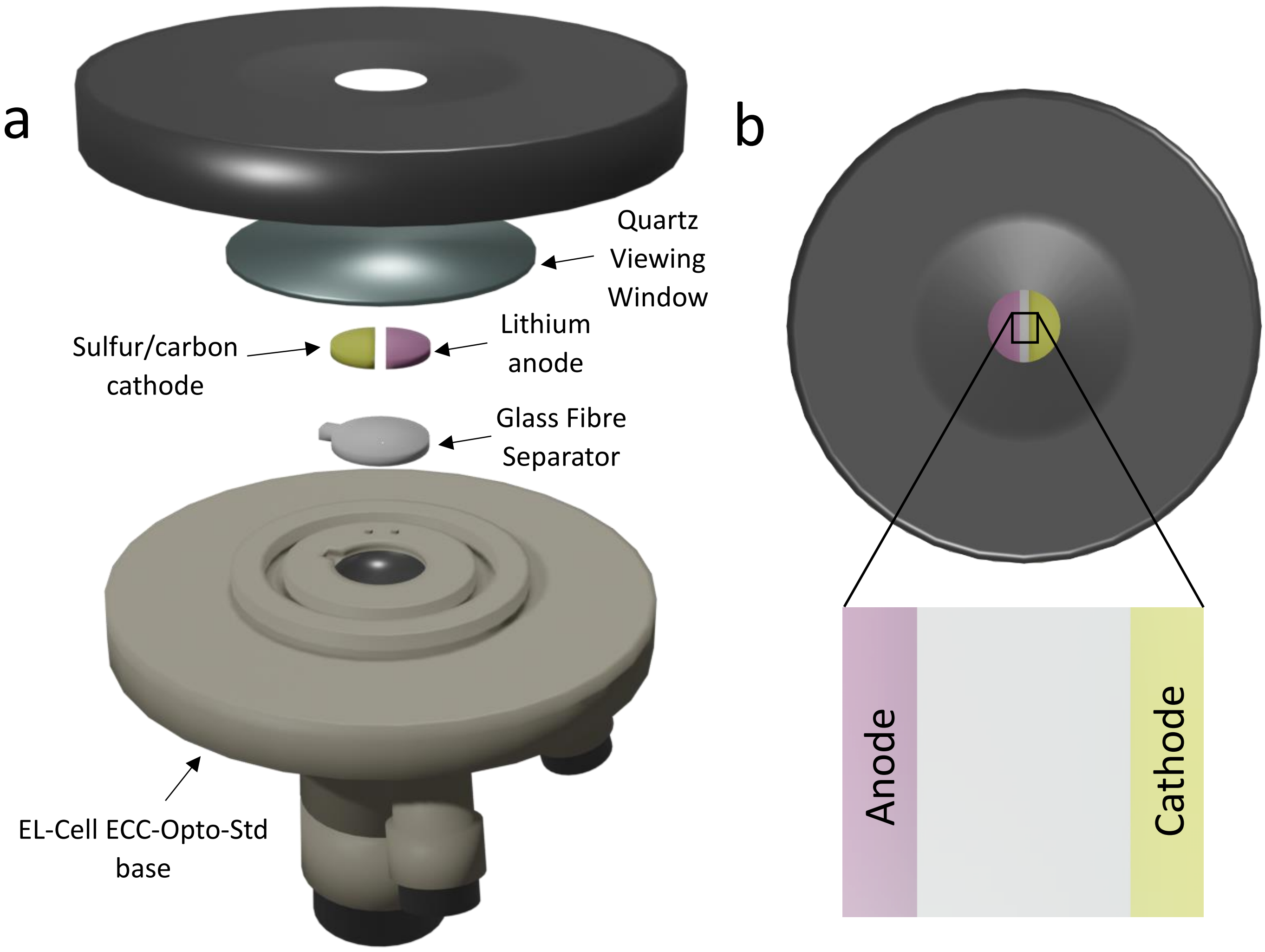

Figure S6: (a) Blown up schematic of the EL-Cell ECC-Opto-Std, showing the arrangement of semi-circular electrodes under the viewing window. (b) Depiction of the view through the viewing window of the constructed optical microscopy cell. The two electrodes are clearly framed alongside a region of electrolyte. The expansion shows the approximate area imaged by the fluorescence microscope during the experiments.

S7. Schematic illustration of the test principle of the optical fluorescence microscopy technique

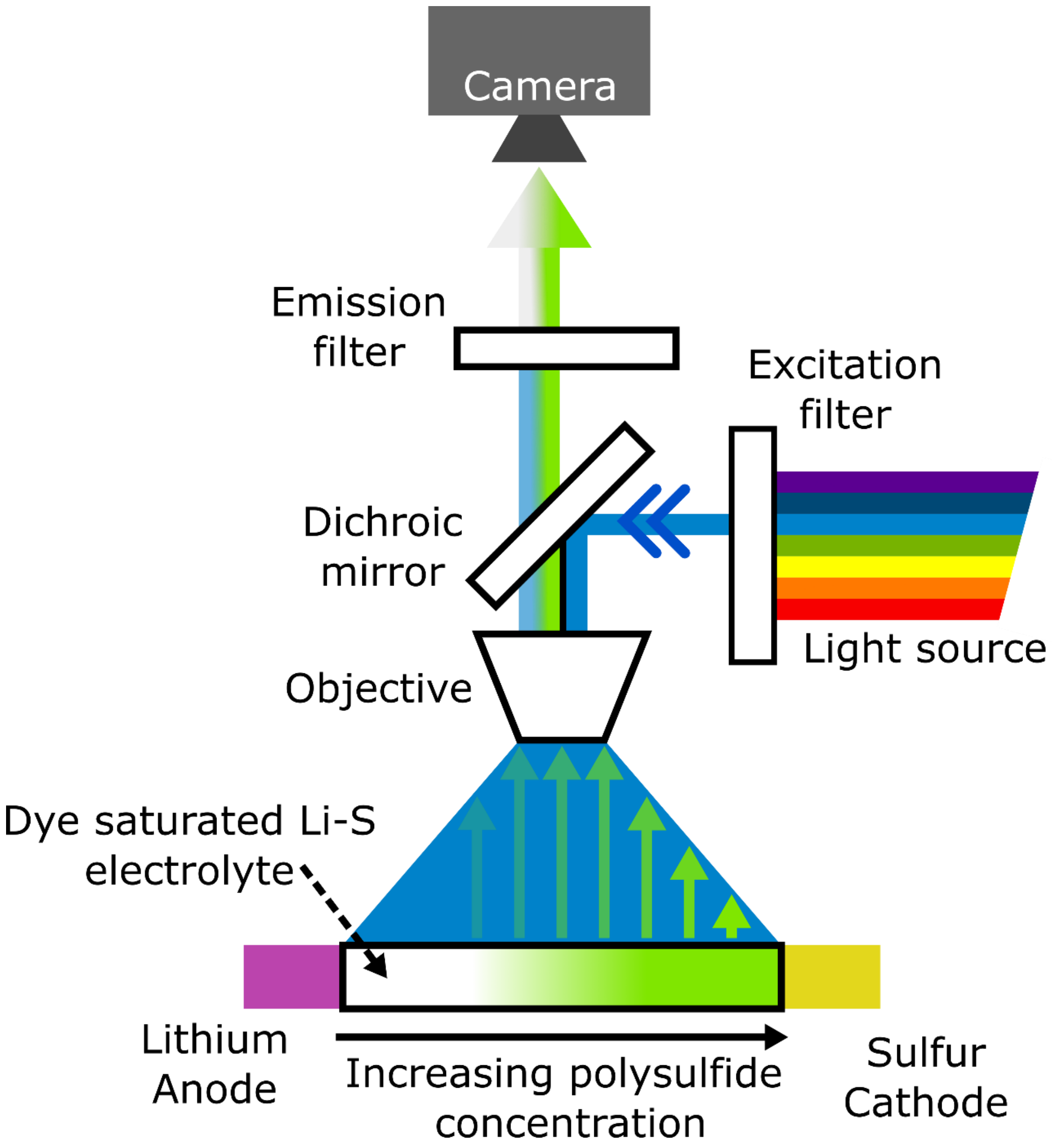

Figure S7: Schematic illustration of the test principle of the optical fluorescence microscopy technique. Light at the excitation wavelength for the dye is shone onto the dye saturated electrolyte, and the intensity of emitted fluorescence detected by the camera is proportional to the polysulfide concentration.

S8. UV-Vis of PS-Li_2S_x with and without the addition of polysulfides

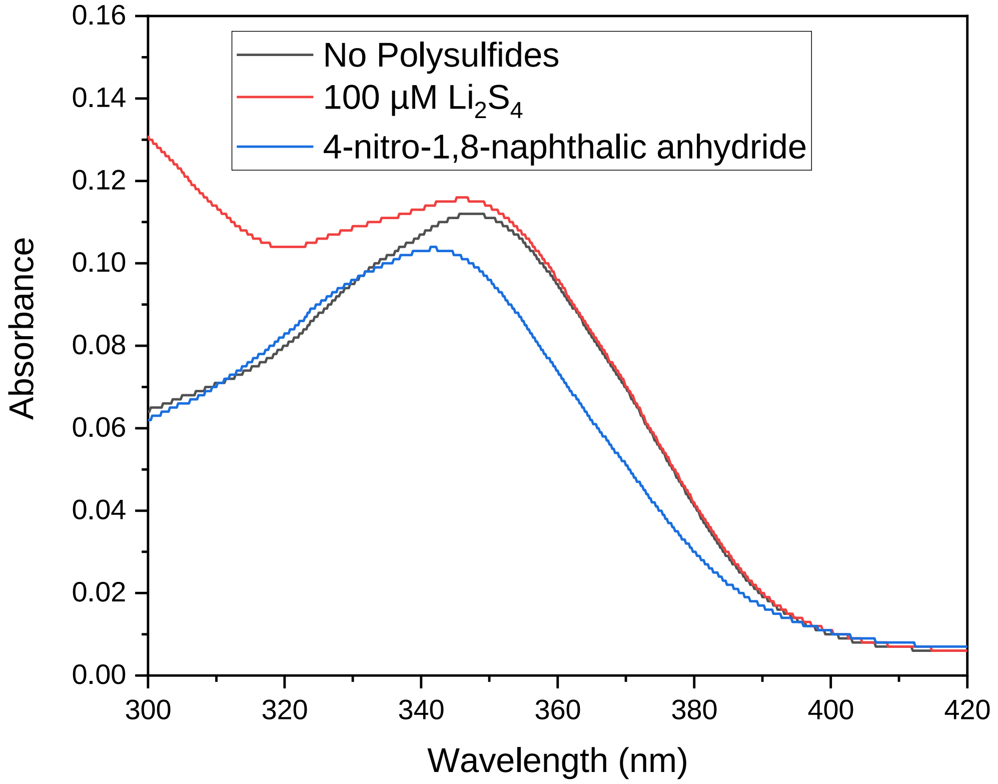

Figure S8: UV Absorption spectra for 10 µM of the polysulfide sensitive dye with and without the addition of 100 µM of Li2S4, alongside 10 µM of the dye precursor 4-nitro-1,8-naphthalic anhydride

S9. Example images demonstrating PS-Li_2S_x fluorescence selectivity

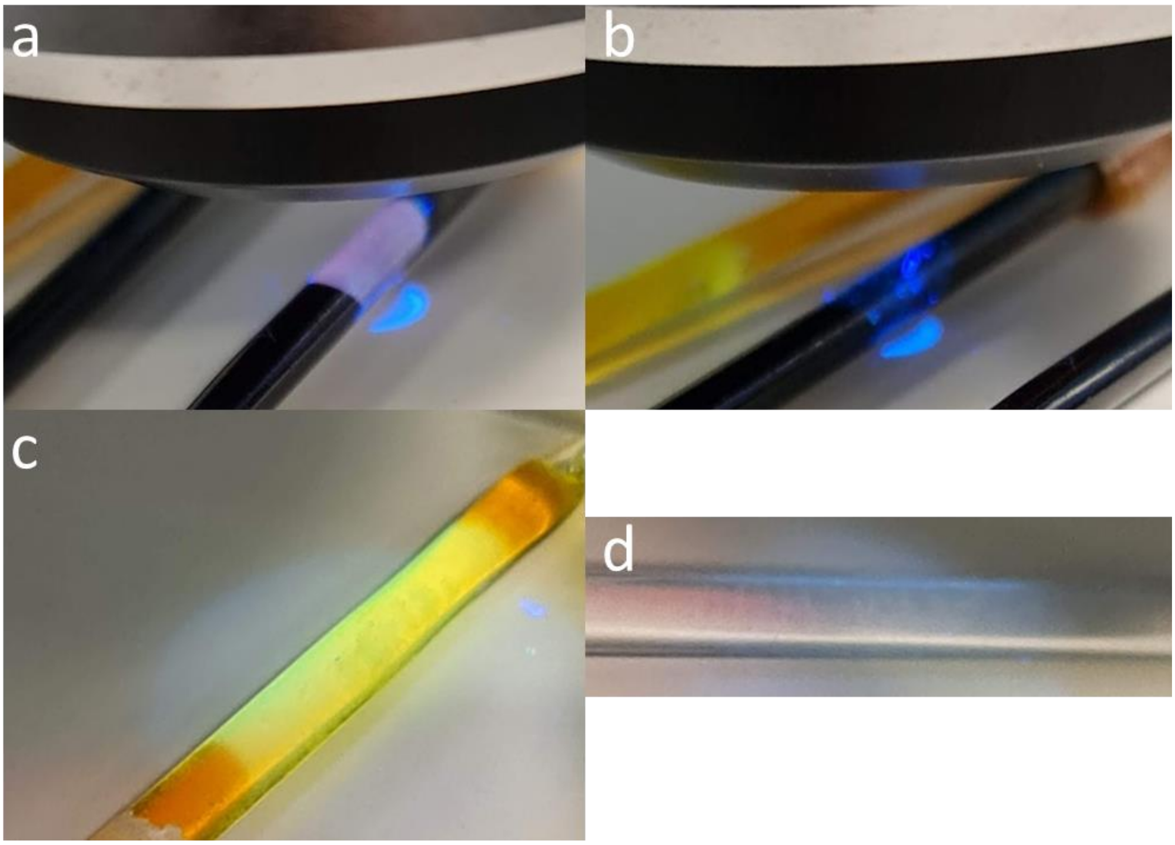

Figure S9: Capillaries under 470 nm fluorescent light containing (a) 0.1 M Li_2S_4 and 1 M PS-Li_2S_x in methanol (b) 0.1 M Li_2S_4 in methanol (c) 1 M LiTFSI and 0.8 M $LiNO_3$ in (1:1 v) DOL:DME (Standard LiSTAR electrolyte) and 1 M PS-Li_2S_x (d) 1 M LiTFSI and 0.8 M $LiNO_3$ in (1:1 v) DOL:DME (Standard LiSTAR electrolyte). Notably, the Li_2S_4 found in both (a) and (b) only produces a fluorescence response in the presence of (b), while the electrolyte was not fluorescence with or without PS-Li_2S_x.

S10. Calibration curve of PS-Li_2S_x fluorescence intensity against polysulfide concentration

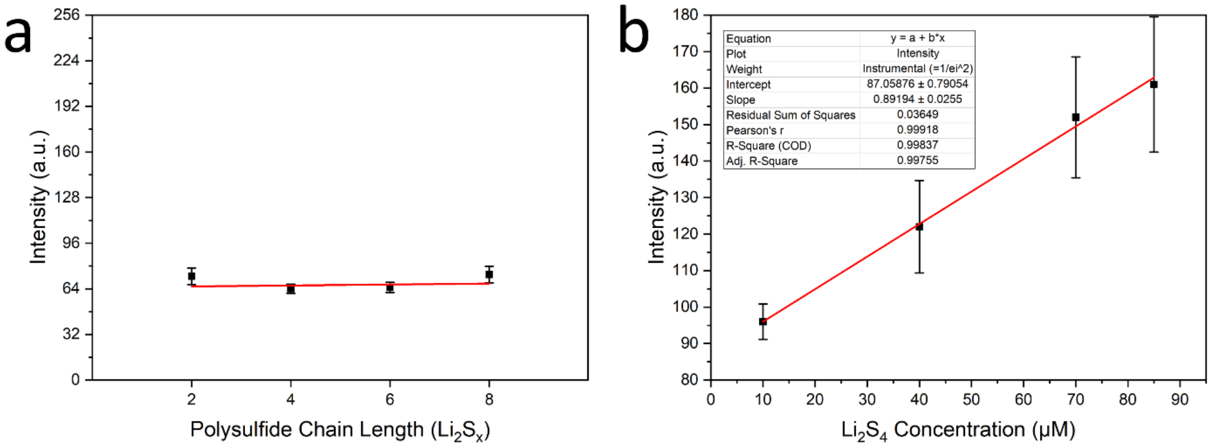

Figure S10: Optical fluorescence measurements taken to correlate optical fluorescence intensity with polysulfide concentration. Measurements taken with the camera settings of 75x zoom and a 500 ms exposure, imaging a glass fibre separator saturated with (a) 40 μM of Li_2S_x (x = 2, 4, 6, 8) (b) the given concentration of Li_2S_4, in 1:1 (v:v) DOL:DME.

S11. *Operando* optical fluorescence microscopy study of a standard Li-S cell showing cycling data and fluorescence intensity for all cycles

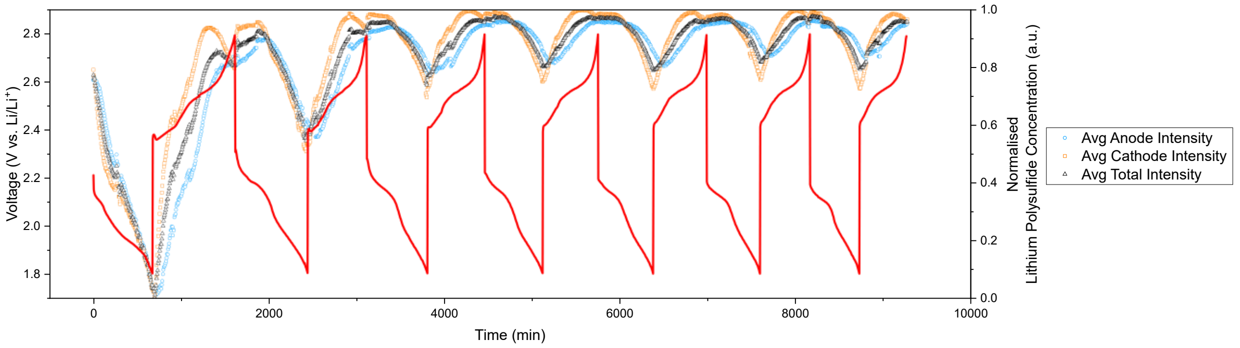

Figure S11: Cycling data and normalised lithium polysulfide concentration, derived from average fluorescence intensity values, taken during operando study of the electrolyte of a standard Li-S cell, with 20 µM of the polysulfide sensitive PS-Li_2S_x fluorescent dye

S12. *Operando* optical fluorescence microscopy study of an Li-S cell without the $LiNO_3$ additive

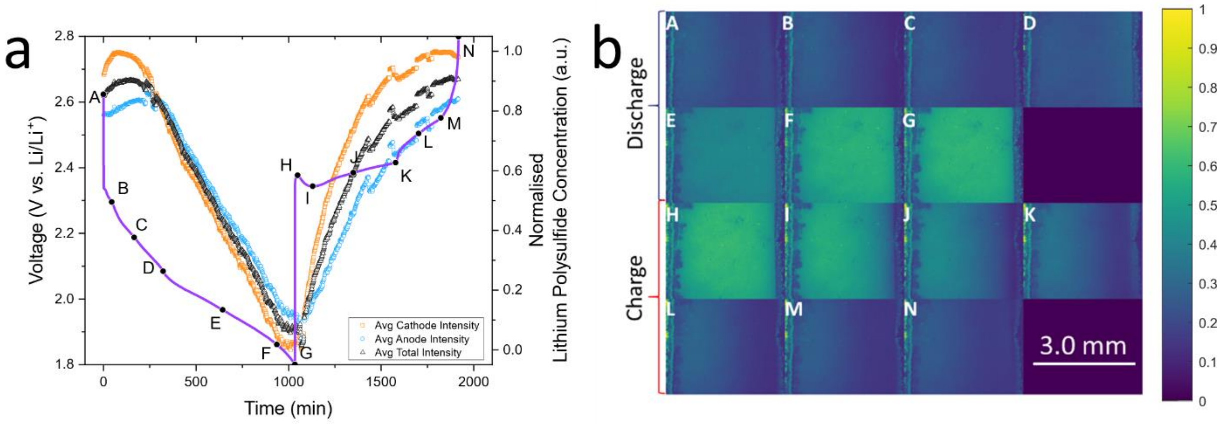

Figure S12: Optical fluorescence images (b) and cycling data (a) taken during operando study of the electrolyte of a standard Li-S cell, omitting the SEI protective $LiNO_3$ additive, with 20 µM of the polysulfide sensitive PS-Li_2S_x fluorescent dye for the second cycle.

S13. *Operando* optical fluorescence microscopy study of an Li-S cell without the $LiNO_3$ additive, showing cycling data and fluorescence intensity for all cycles

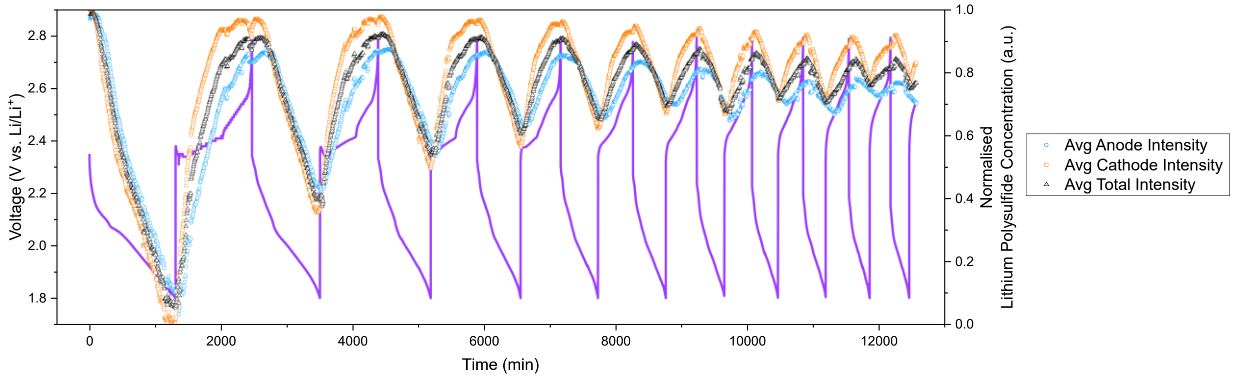

Figure S13: Cycling data and normalised lithium polysulfide concentration, derived from average fluorescence intensity values taken during operando study of the electrolyte of a standard Li-S cell, omitting the SEI protective $LiNO_3$ additive, with 20 µM of the polysulfide sensitive PS-Li_2S_x fluorescent dye

S14. Optical microscopy confocal depth profiling of surface pores prominence within the NEI cathode

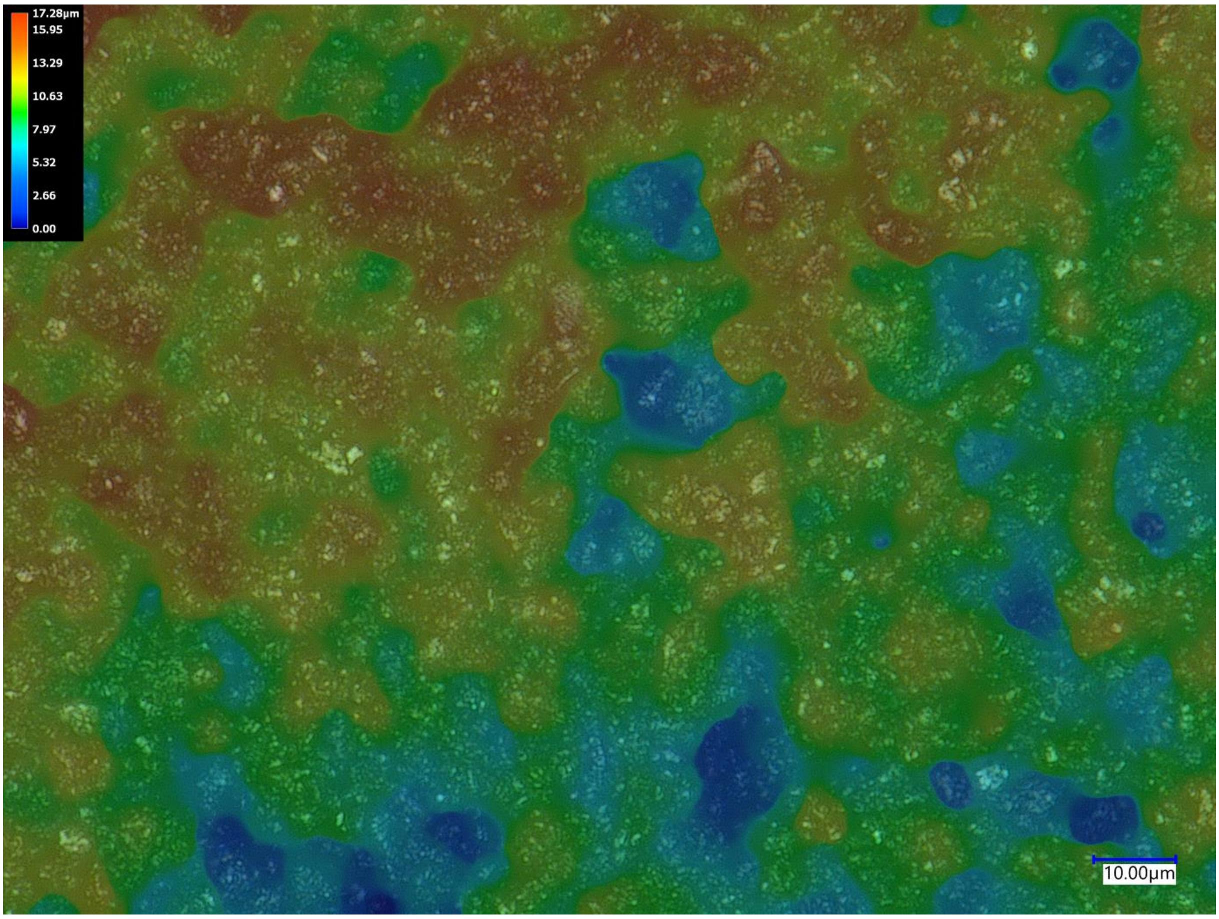

Figure S14: Optical microscopy confocal depth profiling of surface pores within the NEI Nanomyte BE-70 cathode
